# Profile: Stigma and the psychiatrist - Julia Bland talks to Dinesh Bhugra

**DOI:** 10.1192/pb.bp.114.048520

**Published:** 2014-08

**Authors:** Julia Bland

Professor Dinesh Bhugra made bold attempts to move psychiatry forward when President of the Royal College of Psychiatrists. In May 2012, Julia Bland went to pick his brains as he prepares to take up the post as head of the World Psychiatric Association (WPA).

It is a sign of how things have changed when an Indian-born, openly gay man is elected to lead a profession which up until relatively recently classified homosexuality as a disease. But it is apparent that the newly appointed president of the World Psychiatric Association will have his work cut out. In Uganda a recent law has introduced draconian punishments for homosexuality. In India the president of the Indian Psychiatric Society threw her hands up in horror and said, ‘We don’t talk about that here!’ It is a good job then that Professor Bhugra’s achievements and experience are immense and having practised psychiatry for more than 30 years, it would be fair to say he is probably the best person for the post.

He adopts a progressive agenda, having published and spoken out for beleaguered patients, overseas doctors, women and lesbian, gay, bisexual and transgender (LGBT) people for many years. The 61-year-old describes himself as an ‘optimist’, laughing apologetically, as if the concept of optimism somehow excuses the gap between noble aspiration and reality. ‘We have to take a stand’ is his rallying cry to the profession. ‘If we don’t, we are sleepwalking over a cliff’, he added. He believes unity of voice is crucial. ‘If we are seen as divided as a profession, policy makers will take the opportunity to divide and rule’, he said. He dislikes the old dreary and pointless internal squabbles between biological and social psychiatry.

## Psychiatrists’ mental health

He is exercised about the health and well-being of doctors - and especially psychiatrists. He described an interesting consultation the College sent out to over 7000 consultant psychiatrists in 2010, which he presented at the time. The results of the survey were not published since it was felt to be of poor quality.

**Figure:**
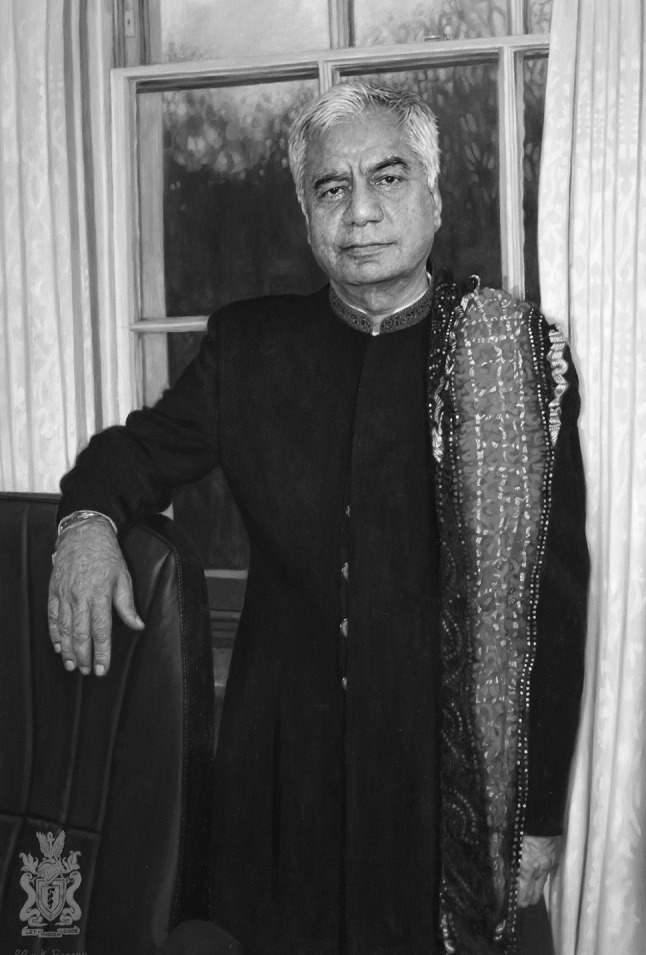


Professor Bhugra sees self-disclosure, ‘coming out’ as mentally ill, as a personal choice, but very different from ‘coming out’ as gay: ‘One can hide sexuality but may not be able to hide mental illness.’ But he agrees that self-disclosure of mental illness may reduce stigma (Ruby Wax, Stephen Fry, and past president of the College, Mike Shooter, to name a few of the people who have worked to reduce stigma in this way). He thinks doctors need to learn to look after themselves and welcomes the idea of self-reflective practice groups at all stages, including at consultant level, and across specialties.

‘If doctors understand the importance of the public mental health agenda as it applies to themselves, things like exercise, meditation and mentalisation, they are less likely to lose their humanity.’ And he recommends that the College might repeat the 2010 survey.

## Service provision

He agreed that the preventative mental health message is getting lost (for example, £1 spent on child psychiatric services is estimated to save £6 in later health, forensic and social costs). With regard to the services provided for patients, the Health and Social Care Act 2012 with its attendant re-commissioning of services in the direction of the cheap and cheerful was at least partly to blame for falling standards. ‘Only forensic psychiatry is thriving’, he says with more than a hint of irony, and ‘child psychiatry is being swallowed up into community paediatrics in some areas’. He suggests that the ‘conversation’ needs to be with the public mental health lead within Public Health England, a separate body from NHS England.

## Frustration

Although he is discreet and measured in his language, there is an undercurrent of frustration at stymied initiatives from his period as president of the Royal College of Psychiatrists. His attempts to encourage ‘youth psychiatry’ for the important 14- to 24-year-old group met with protectionist resistance. ‘People feel more comfortable where they are’, was his comment on the conservatism and parochialism of the profession, but his irritation was audible. He made the point that when psychiatrists feel devalued, they should engage patients, their families and general practitioners (GPs) as advocates: ‘GPs want their patients assessed initially by a psychiatrist, not by a second-year nurse. And families feel the same about their sick family members. This is no disrespect to multidisciplinary teams, just about training and experience’, he said.

He has also been frustrated by inflexible psychiatric colleagues, citing the obvious benefits of community mental health teams (CMHTs) being located in primary care: collaboration, cross-referral, more seamless care for patients. It was the psychiatrist who refused to join a primary care centre, when the GPs were ready to welcome them, in one recent instance.

When he started as College president he described the lamentable state of many in-patient wards, which hit the front pages. This was welcomed by patients but ‘psychiatrists hated it, feeling criticised’. Interesting to consider the duty to whistleblow in the post-Francis era.

## Training

Dinesh Bhugra also has strong views about humanity getting lost in medical training, a characteristic that has to be retained to be a good psychiatrist, in his view. ‘What we do now is take the brightest students as medics, drill competition in and drill empathy out. Then we expect them to emerge as team players!If I could change the world, I’d have all the disciplines, medical, nursing, psychology, doing a first year of humanities together, learning anthropology, sociology and literature, before they disappear into their separate silos’, he said. ‘We need psychiatrists who can put themselves in their patients’ shoes, not those whose instinct is to hide behind professionalism. I teach medical students to think about the patient rather than the symptoms’, he added. ‘Young doctors need to see psychiatry as the most interesting and exciting branch of medicine, and to look after their own mental health from the beginning of their training. If we turn the younger generation into sausage packers, delivering commodified packets of care, we will have let them down.’

## Culture and personal life

In an interview with *The Guardian*,^1^ he spoke publicly for the first time about his personal life and sexuality. Growing up in northern India, there were constraining cultural expectations. Moving to the UK in the 1970s made life easier, although he identifies with the difficulties of integration experienced by foreign medical graduates. As a former General Medical Council assessor of poorly performing doctors, he knows the awkward fact that ‘four or five out of six such doctors are from ethnic minorities’. And as chair of the College’s Overseas Doctors’ Training Committee for 6 years, he remembers that the most common complaint against these doctors was their culturally inappropriate request to a nurse to ‘make me a cup of tea’. Personally, Dinesh Bhugra is an urbane international operator who can play comfortably with his own multiple identities as he jets around the globe, but he fully recognises the complexity around teasing out real poor performance from the experience of discrimination for overseas medical graduates.

He regrets the ending of an imaginative College induction course for overseas doctors, and as we discussed its demise for financial reasons, he decided to write to the diaspora organisations to remind them to look after their own.

It seems, then that Professor Bhugra is not afraid to speak out or indeed to stand out from the crowd. His portrait in the Royal College of Psychiatrists is not an identikit of a suit, shirt and tie. He is dressed in full traditional Indian regalia appearing like a contemporary maharajah surveying his kingdom.

He lives in Brixton with Mike, his partner of more than 30 years, and maintains an office in the Institute of Psychiatry. Sophisticated, smooth and realistic, his message is forward looking and crystal clear: be creative and flexible, work across boundaries, wake up to the new commissioning realities, don’t be narrow and protectionist - or prepare to be sidelined.

The visionary Bhugra is soon to have an even wider purlieu: the world, when he takes the presidential reins of the WPA this September. He hopes to combine his ambition with realism. He said his priorities will include domestic violence, child abuse, prisoner mental healthcare, minorities including people with intellectual difficulties, LGBT and mental health promotion. Enough to make mere mortals giddy at just the thought of what this might imply. But Bhugra knows the WPA can only be a facilitator, potentially useful as a research hub, connecting people. He said: ‘Psychiatry does not have the answers to everything but we can find partners, make links and at least make a stand.’

He takes a properly global view of mental health and refuses to assume an attitude which smacks of colonial superiority: ‘The tragedy of western psychiatry is that we have been so egocentric, when large swathes of the world are still sociocentric, and we in the West need to learn from other societies. Why aren’t Russian or Asian psychiatric textbooks translated into English? Then we might actually learn from them.’

He has seen wonderfully creative solutions abroad with limited resources: the psychiatric hospital in India where there is such a shortage of nursing staff that patients are only admitted with relatives. The relative becomes the informed co-therapist who can monitor the patient after discharge and becomes an educated participant in treatment. Or the school in Pakistan where children are taught to recognise psychosis and epilepsy. They tell their teacher, who then contacts the health professional resource.

Professor Bhugra is a grown up. He is not throwing his toys out of the pram. He is quietly but firmly reiterating the wise, collaborative and creative way forward, and his lack of stridency enhances the appeal of his message. Will the rest of us manage to take up his challenge or are we herding ourselves, lemming like, towards the cliff edge?
